# Investigation of the vaginal microbiota of dairy cows through genetic sequencing of short (Illumina) and long (PacBio) reads and associations with gestational status

**DOI:** 10.1371/journal.pone.0290026

**Published:** 2023-08-23

**Authors:** Anne Kemmer Souza, Amanda Fonseca Zangirolamo, Ricardo Guella Droher, Francieli Gesleine Capote Bonato, Amauri A. Alfieri, Márcio Carvalho da Costa, Marcelo Marcondes Seneda

**Affiliations:** 1 National Institute of Science and Technology for Dairy Production Chain (INCT–LEITE), Universidade Estadual de Londrina, Londrina, Paraná, Brazil; 2 Laboratory of Biotechnology of Animal Reproduction, Department of Veterinary Clinics, Center for Agricultural Sciences, Universidade Estadual de Londrina, Londrina, Paraná, Brazil; 3 Département de Biomédecine Vétérinaire, University of Montreal, Montreal, Canada; Washington State University - Spokane, UNITED STATES

## Abstract

The vaginal microbiota has been shown to be important in local immune regulation and may play a role in reproduction and fertility. Next-generation sequencing (NGS) technologies have been used to characterize the bovine vaginal microbiota, mainly using short-read sequencing (Illumina). However, the main limitation of this technique is its inability to classify bacteria at the species level. The objective of this study was to characterize the bovine vaginal microbiota at the species level using long-read sequencing (PacBio) and to compare it with the results of short-read sequencing. In addition, the vaginal microbiota of cows that became pregnant after artificial insemination (AI) was compared with that of infertile animals. Thirteen Holstein cows had vaginal swabs collected prior to AI. DNA was extracted and subjected to Illumina and PacBio sequencing to characterize the V4 region and the entire 16S rRNA gene, respectively. PacBio sequencing yielded 366,509 reads that were assigned to 476 species from 27 phyla. However, none of the most abundant reads (>1%) could be classified at the species level. Illumina sequencing yielded more reads and consequently was able to detect a more observed species, but PacBio sequencing was able to detect more unique and rare species. The composition of the vaginal microbiota varies according to the sequencing method used, which might complicate the interpretation of results obtained in the majority of the current studies. The present study expands on the current knowledge of bovine microbiota, highlighting the need for further efforts to improve the current databanks.

## Introduction

Next-generation sequencing (NGS) has recently become a standard method for studying the microbiota in humans and animals. Although most studies have focused on intestinal microbiota, vaginal bacteria have been shown to play important roles in immune regulation and protection against vaginosis [1].

In cattle, knowledge of the composition of the vaginal microbiota was limited until recently because only qualitative descriptive studies using culture-dependent techniques were available [2]. After the development of NGS, unprecedented information regarding the profile of bacterial communities in the bovine reproductive tract related to health and reproductive diseases has been obtained [3–5].

DNA sequencing allows for a broader identification of microorganisms and evaluation of bacterial dynamics and their interactions with the environment [6]. It has also increased the number of sequenced genes and genomes [7, 8], revealing many bacterial species that have not been described before. Illumina technology is responsible for generating >90% of the known sequencing data and has been considered the standard because of its high reliability and low cost [9]. This technology minimizes the time and cost of sequencing by performing short reads (75–600 base pairs at a time), covering only small and specific regions of the 16S rRNA gene, which is most often used for taxonomic classification. Thus, this technology does not allow for classification at the species level. Conversely, NGS platforms with the capacity to read long DNA sequences can sequence the full-length 16S rRNA gene (1500 bp), providing accurate identification at the species level [8].

Long-reading NGS platforms have contributed to the study of microbial diversity from a deeper perspective [10] and evaluating the agreement between the results of both technologies is necessary for better interpretation of results obtained from Illumina studies [11]. The PacBio platform (Pacific Biosciences) is a technology that produces high-fidelity reads of complete 16S rRNA genes, improves the sensitivity and specificity of the taxonomic profile, and reduces the risk of misclassified reads [10, 12]. To date, no study has used long-read sequencing to characterize the vaginal microbiota of cows. A single bacterial genus can contain hundreds of species; therefore, species-level analysis can help identify biomarkers more precisely that can later be used as diagnostic tools for the prediction and prevention of diseases [13].

There is evidence suggesting that infertile women have a different microbiota profile than fertile women [14–16]. Kyono et al. (2018) [17] observed that a high abundance of *Lactobacillus* spp. may improve the implantation rate, especially in women undergoing fertilization, and the restoration of normal vaginal microbiota to enhance reproduction [18]. Furthermore, studies have shown that *Lactobacillus vaginalis* is positively correlated with the pregnancy rate [19], and its abundance is reduced in women who have repeated failure to conceive [1].

Currently, microbiota studies emphasize the pathogenicity of bacteria; however, by understanding the interaction between the host environment and its microorganisms in greater depth, it is possible to identify biological markers that are beneficial to the host. Therefore, the objective of this study was to characterize the bovine vaginal microbiota at the species level using long-read sequencing (PacBio) and to compare it with results of short-read sequencing (Illumina). In addition, the vaginal microbiota of cows that became pregnant after artificial insemination (AI) was compared with that of infertile animals.

## Materials and methods

### Ethics committee statement

This study was approved by the Institutional Animal Use Ethics Committee (CEUA) of the Universidade Estadual de Londrina (UEL) under protocol number 10878.2019.88, on August 6, 2019.

### Animals

Thirteen Holstein Friesian cows with a mean age of 5 ± 1.3 years housed at a dairy farm (24° 47′ 32″ south, 50° 0′ 42″ west) were evaluated. The selected cows had high milk production (daily average of 42.2 ± 10.09 liters of milk), were multiparous, ranging from the second to the seventh calving (average of three births), and were not subjected to a hormonal protocol for synchronization and ovulation induction. The animals had a reproductive history free of abnormalities and were not treated with antibiotics. The cows were milked twice daily and subjected to a semi-confinement system. The animals spent part of the day in paddocks with Azevém (*Lolium multiflorum*) pasture and received a diet in the trough based on commercial concentrate (16% crude protein) and corn silage after each milking. All females were healthy and had an up-to-date vaccination status for reproductive diseases.

### Reproductive management

Estrus detection was performed using a monitoring collar (Allflex Company, Livestock Intelligence, Joinville, Santa Catarina, Brazil). On the day of estrus, the cows were inseminated following reproductive management process adopted on the farm. Pregnancy was diagnosed 30 d after insemination using ultrasonography with a 5 MHz linear rectal probe (Kaxin device model KX5200, Jiangsu, China). Cows were classified as pregnant (PG) or non-pregnant (NP) and remained with the rest of the herd throughout the experiment, with no changes in reproductive, sanitary, food, and zootechnical management.

### Sample collection and DNA extraction

A vaginal sample from each cow was collected before AI using a sterile swab introduced into the caudal portion of the vagina. To avoid contamination, the perineal region was cleaned with a disposable paper towel soaked in 70% alcohol. The vulvar lips were opened using clean gloves, and the swab was introduced by performing 15 circulatory movements touching the vaginal mucosa. Subsequently, the swab shaft was cut and the tip was placed in a sterile microtube. The samples were collected in duplicate and immediately placed in a liquid nitrogen cylinder for transport to the Laboratory of Animal Reproduction Biotechnology (REPROA) at the Universidade Estadual de Londrina, Londrina, PR, Brazil. For the extraction of genetic material, a commercial PowerSoil® kit (QIAGEN, Hilden, Germany) was used, following the manufacturer’s specifications, then the samples were stored at -20°C. A sterile swab was included in the DNA extraction protocol to be used as negative control to account for environmental contamination, which contained no good quality reads after PCR amplification.

### Next-generation sequencing and bioinformatics

The extracted DNA was subjected to two different analyses: the first using short reads (Illumina) and the second using long reads (PacBio).

For short-read sequencing, the V4 region of the bacterial 16S rRNA gene was amplified using polymerase chain reaction (PCR) for sequencing using Illumina with primers 515F (5’-GTGCCAGCMGCCGCGGTAA-3’) and 806R (5’-GGACTACHVGGGTWTCTAAT-3’). Amplicons were sequenced with the Illumina MiSeq platform (San Diego, California, United States) using the V2 reagent kit (2 × 250 cycles = 500 base pairs) at the Genome Québec Innovation Center, McGill University, Montreal, QC, Canada. Bioinformatics analyses were performed using Mothur software v.1.41.3 [20], following the Standard Operating Procedure [21]. Low-quality sequences were excluded, and high-quality reads were aligned with the SILVA reference database [22]. The reads were combined into inference amplicon sequence variants (ASVs) and classified using the Ribosomal Database Project (RDP, update from July 2020) [23].

For long-read sequencing, the DNA was submitted to the Center for Genotyping and Sequencing, Delaware Institute of Biotechnology, University of Delaware, Newark, DE, United States. The full-length 16S rRNA gene was amplified using PCR with the following primers 27f: 5’-AGRRTTYGATYHTDGYTYAG-3’ forward, and 1492r: 5’-TASVGHTACCTTGTTACCGACTT-3’ reverse. Amplicon libraries were created using the SMRTbell Express Template Prep kit 2.0 (Pacific Biosciences), according to the manufacturer’s instructions and sequenced on a Sequel 2 system (Pacific Biosciences). In addition, further analysis previously recommended to classify sequences at the species level [24] was performed using DADA2 software [25] for the detection of sequencing errors and for grouping the reads into ASVs for further taxonomic assignment. SBanalyzer 2.4 software (Shoreline Biome) was used to map circular consensus reads (CCS) to the Athena database and assign taxonomic identification to all reads [24].

For the comparison between methods (Illumina versus PacBio), the same parameters used for short-read analysis were used in mothur, except for the length of the reads that were set to a minimum of 1500 bp.

### Alpha and beta diversity

Results from short- and long-read sequencing were both used for alpha and beta diversity calculations using Mothur software. Alpha diversity was characterized by the total number of species observed, the Chao index (richness estimator), the Simpson diversity index, and the Shannon diversity index. Beta diversity (comparison between the communities) was determined using the Jaccard index, considering only the taxonomic composition of the community (presence or absence of each species), and the Bray-Curtis index, which also considers the distribution of each species (relative abundances). Principal Coordinate Analysis (PCoA) was used to visualize similarities between the samples analyzed independently using Illumina and PacBio.

### Statistical analysis

The Minitab 18 software was used to compare the different metrics obtained from short- and long-read sequencing. Data are expressed as mean ± SD, unless otherwise indicated. A t test was used to compare the relative abundances of the phyla found by the different platforms (Illumina and PacBio) as well as to compare alpha diversity indices and the Bonferroni test subsequently applied for multiple comparison correction. The correlation between alpha diversity indices (number of genera; Chao, Simpson, and Shannon) obtained using the two methodologies (Illumina and PacBio) was determined using the Pearson test (p≤0.05).

## Results

### Characterization of the bovine vaginal microbiota at the species level

Sequencing of the whole 16S rRNA gene using long-read PacBio technology from vaginal samples collected from 13 Holsten cows yielded 366,509 sequences. These were assigned to 476 species from 22 phyla. However, none of the most abundant reads (>1%) could be classified at species level.

**Fig 1:**
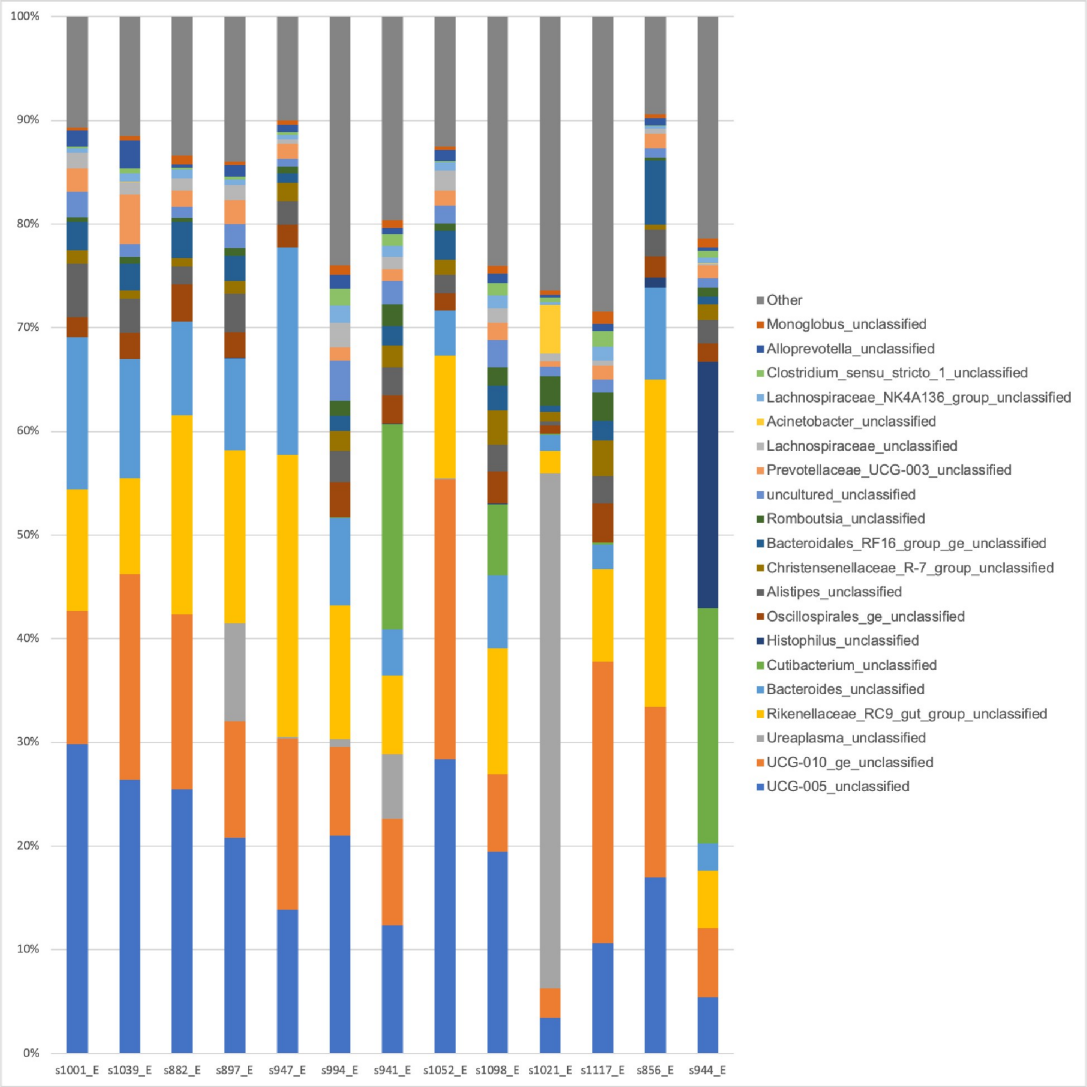
Relative abundance of the 15 most abundant species of bacteria found in the vaginal microbiota of Holstein cows, identified using long-read sequencing (PacBio platform).

The 15 most abundant bacterial species are shown in Fig 1. Approximately 51% of the vaginal microbiota was represented by five species: *UCG-005_*unclassified (from the Oscillospiraceae), *UCG-010_ge_unclassified* (from the Oscillospiraceae family), *Ureaplasma_*unclassified, *Rikenellaceae_RC9_*gut_group_unclassified, and *Bacteroides_*unclassified.

### Comparison of NGS methodologies (Illumina *vs*. PacBio)

**Fig 2:**
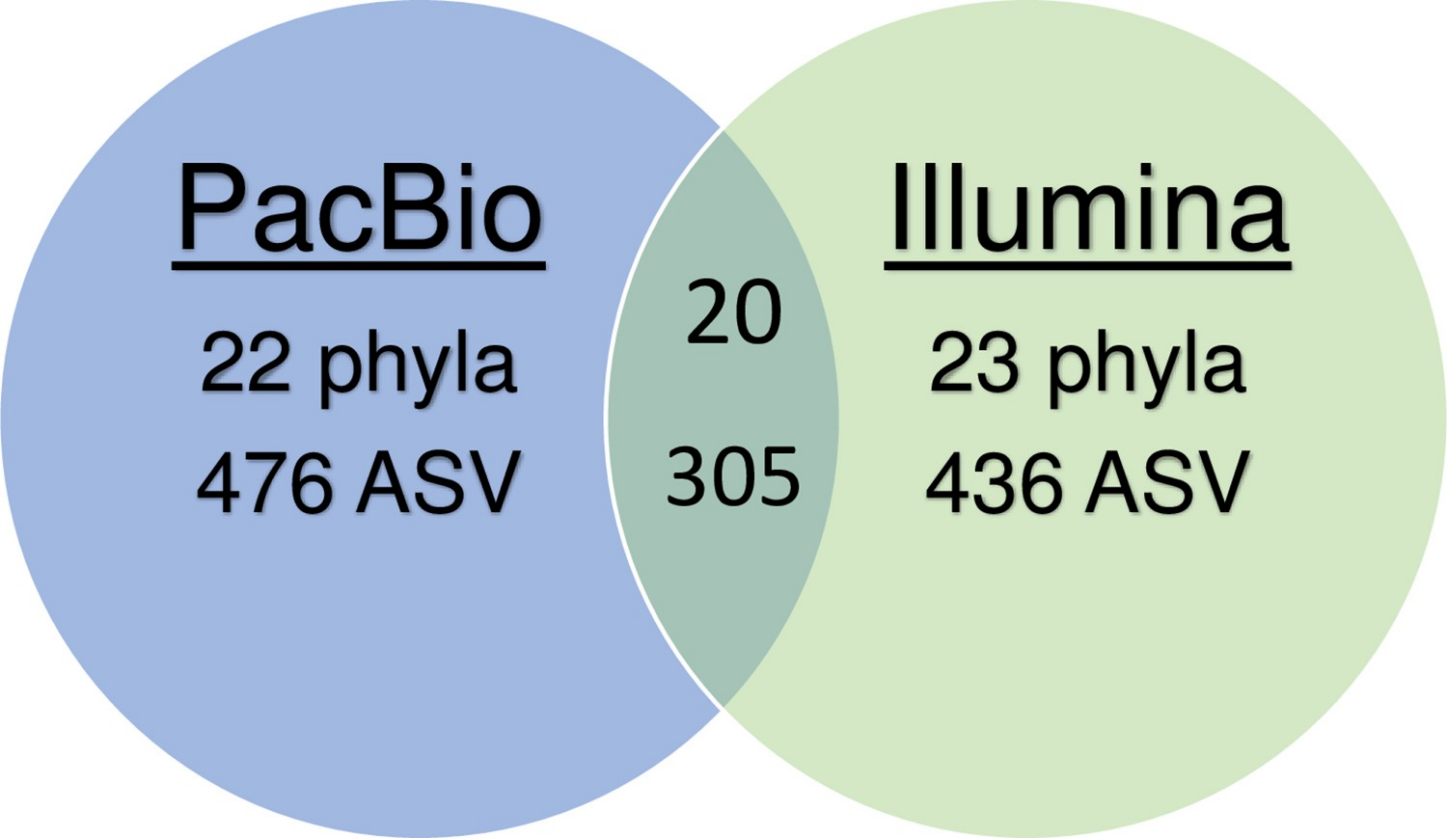
Venn diagram representing the number of unique and shared phyla and ASVs between the PacBio and Illumina NGS technologies.

Sequencing of the V4 region of the 16S rRNA gene using the Illumina platform yielded 631,773 good-quality reads that were classified into 436 ASVs from 23 phyla, adopting a subsampling of 34,771 reads per sample. Long-read sequencing using the PacBio platform yielded 195,589 good-quality reads that were classified into 476 ASVs from 22 phyla. Although the number of phyla was similar between the two methods, only 20 phyla and 305 ASVs were assigned to the same taxon (Fig 2). Nevertheless, there was an agreement between the five most abundant phyla (Firmicutes, Bacteroidetes, Actinobacteria, Proteobacteria, and unclassified bacteria), as presented in Table 1, which corresponded to 96% of the total reads in the PacBio platform and 97% in the Illumina platform. After Bonferroni correction for multiple comparisons, only unclassified Bacteria were significantly more abundant in Illumina (mean = 7.6% SD = 3.70%) compared to PacBio (mean = 0.6%, SD = 0.55%) (p<0.0001).

**Table 1:** Comparison of the averages of the five most abundant phyla found in the vagina of dairy cows using the PacBio and Illumina platforms.

Taxon	Mean (%) Ilumina	Mean (%) PacBio
*Firmicutes*	50	35
*Bacteroidetes*	26	47
*Actinobacteria*	3	2
*Proteobacteria*	10	8
Unclassified bacteria*	8	1

*Significant difference with 95% confidence interval (p≤0.05)

**Fig 3:**
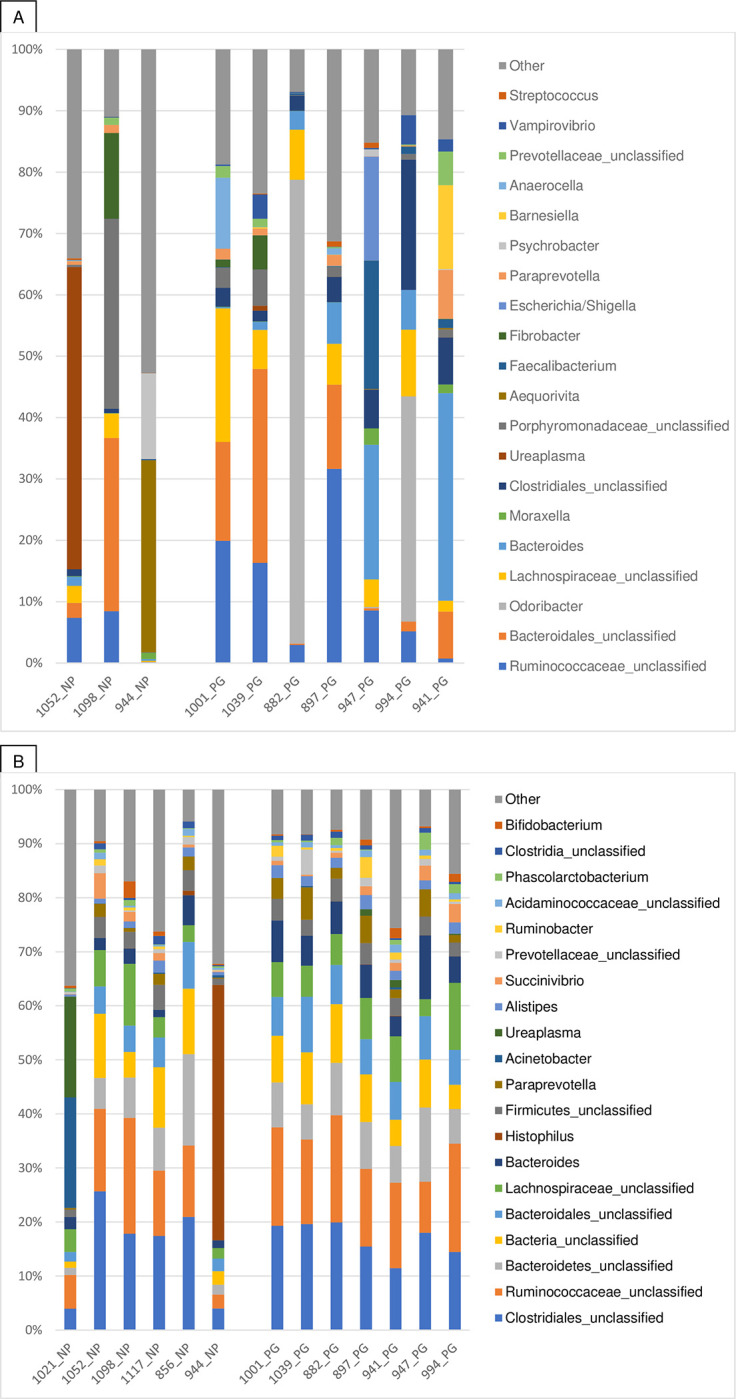
Relative abundance of the 20 most predominant ASVs present in the vaginal microbiota of dairy cows identified using the PacBio (A) and the Illumina platforms (B). Cows that failed to become pregnant after artificial insemination are indicated with NP (non-pregnant) and the ones that became pregnant with PG (pregnant). The order of abundance is represented from bottom to top in the legend.

Fig 3 shows the relative abundance of the 20 most abundant genera identified using PacBio and Illumina sequencing. Although many genera were shared between the two platforms, the figure shows a clear difference between the most abundant bacteria identified in this study.

Alpha diversity was calculated using the number of observed species, the Chao, Simpson, and Shannon indices for each methodology, and compared using analysis of variance (ANOVA). Results of the statistical analysis as well as the mean and standard deviation are presented in (Table 2). There was a significant difference in the number of genera and the Chao index between the different sequencing techniques (p≤0.05), but not for the Simpson and Shannon indices.

**Table 2:** Means, standard deviation, and p-value (≤0.05) of alpha diversity indices present in the vaginal microbiota of dairy cows comparing the Illumina and PacBio sequencing techniques.

	Observed species (ASVs)	Chao	Simpson	Shannon
**Illumina**	10,848 ± 2,488	19,7646 ± 65,672	362.88 ± 207.52	7.48 ± 0.83
**PacBio**	5,128 ± 516	2,082,863 ± 2,394,221	726.89 ± 963.59	8.05 ± 0.46
**p-value**	<0.001*	0.005*	0.099	0. 032

*Significant difference after correction for multiple comparisons.

Subsequently, the correlation between the alpha diversity indices obtained with the two techniques was performed, and there was no statistical difference between the techniques (Table 3). For the correlation test, the alpha diversity values of samples excluded from the PacBio analysis were also excluded from the Illumina analysis.

**Table 3:** Correlation (r) and p-value (≤0.005) of alpha diversity indices present in the vaginal microbiota of dairy cows comparing the Illumina and PacBio sequencing techniques.

	Observed species (ASVs)	Chao	InvSimpson	Shannon
**r**	0.110	-0.082	0.185	0.165
**p-value**	0.762	0.822	0.609	0.648

### Comparison between bacterial communities of cows that became pregnant or not after AI

After AI, cows that did not become pregnant were observed for difficulty in conceiving, which was confirmed by four subsequent inseminations on average to achieve pregnancy (standard deviation ± 2). Of the 13 cows evaluated in this study, seven became pregnant (PG) after AI and six remained non-pregnant (NP).

**Fig 4:**
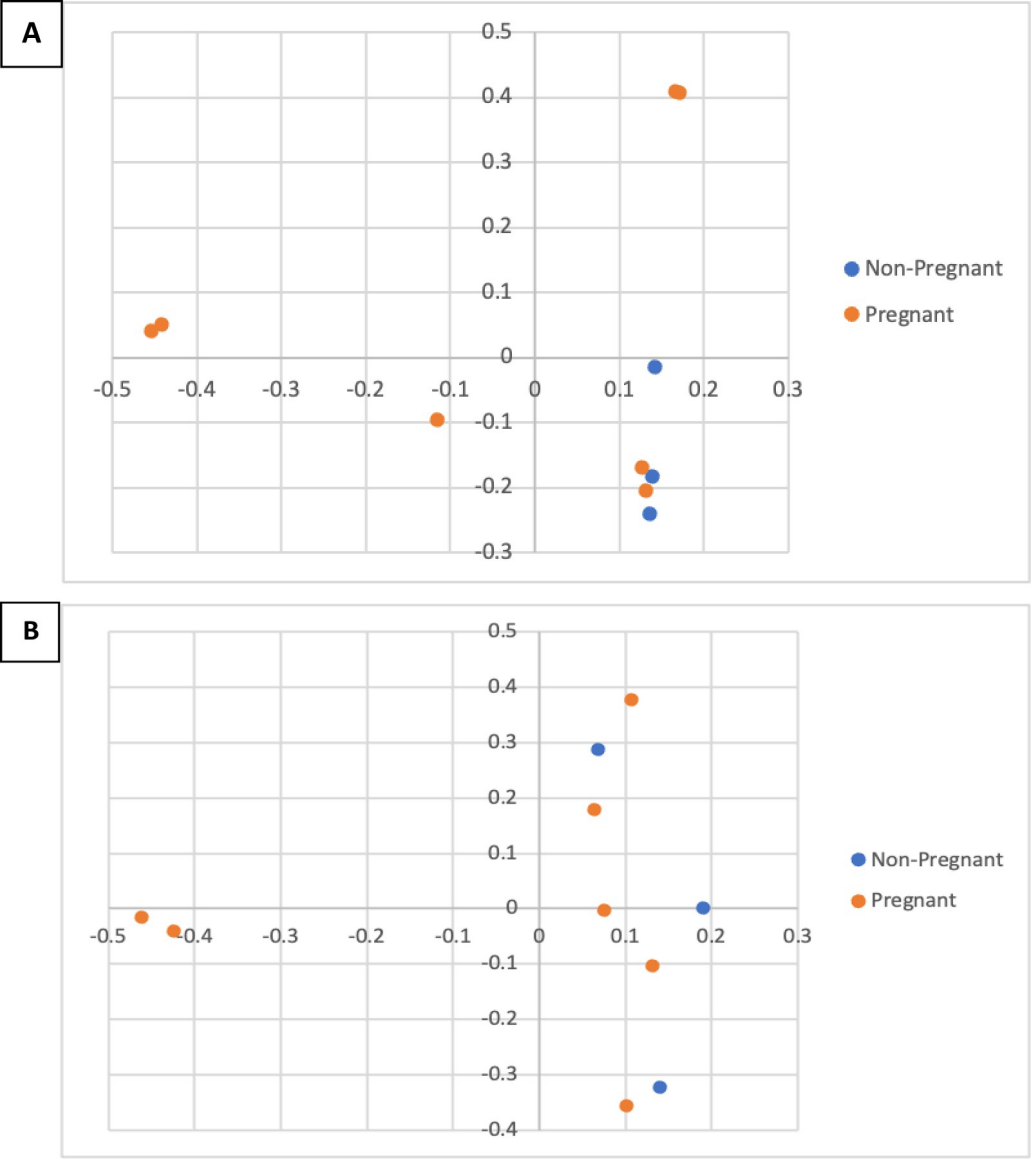
PCoA representing the similarity of the vaginal microbiota structure (A) and membership (B) of pregnant and non-pregnant dairy cows addressed using the Brady-Curtis and the Jaccard index, respectively.

Results from PacBio sequencing were used to compare the similarities among samples (beta diversity) with PCoA using the Bray-Curtis index (considering taxonomic composition and evenness of each taxon), as well as the Jaccard index (considering the presence or absence of each taxon), as shown in Fig 4. There were no statistical differences (AMOVA) between cows that became pregnant and cows that failed to conceive studied with PacBio sequencing in both community structure (Bray-Curtis) and membership (Jaccard) (p = 0.168 and p = 0.350, respectively).

There was no statistical difference in the most abundant genera between PG and NP cows (p≥0.05). It is noteworthy that there was a marked interindividual variation, as illustrated in Fig 3, showing a high relative abundance of *Ureaplasma* (48%) in cow 1021 and *Cutibacterium* in cows 941 (19%) and 944 (22%).

## Discussion

### Investigation of the vaginal microbiota at the species level

This study is the first to report the bacterial species present in the vaginal microbiota of Holstein cows. Short-read sequencing (Illumina) is the major technology currently used for microbiota characterization, but the major limitation of this method is the ability to classify bacteria to lower taxonomic levels (i.e. species). For instance, one analysis may reveal that Ureaplasma is the most abundant bacteria in an environment, but information regarding which species of Ureaplasma are present remains unknown. Conversely, long-read sequencing technologies (PacBio) can classify bacteria at the species-level [26]. However, the PacBio sequencing results obtained from this study were unable to classify the most abundant species found in the bovine vaginal microbiota. This may have occurred because of the genes identified to belong to species not yet known or the inability of the databases used to provide this information.

Interestingly, marked heterogeneity of the vaginal microbiota was observed among the different cows, probably influenced by intrinsic and extrinsic factors in the host, highlighting the need for larger experimental sizes for studies investigating bovine vaginal microbiota. Unclassified *UCG-005*, unclassified *UCG-010*, unclassified *Ureaplasma*, unclassified *Rikenellaceae_RC9*, and unclassified *Bacteroides* were the main taxa found in the 13 cows used in this study. However, little is known about the role of these strains in the reproductive tract of cows. Unclassified *UCG-005* (family Oscillospiraceae, phylum Firmicutes) has been described as the most abundant in dairy cattle in several studies [5, 27–29] and, therefore, may be important in maintaining homeostasis in this environment.

Firmicutes bacteria assist in the process of food digestion as they promote the degradation of fibers and the conversion of cellulose into volatile fatty acids; thus, they are involved in the absorption and production of nutrients, defense against the invasion of foreign pathogens in the gastrointestinal tract, and energetic metabolism of the host [3, 30]. The genus *UCG-005* has been associated with higher resilience of the intestinal microbiota in other species [30], but its role in the vaginal microbiota remains to be investigated. Unclassified *Ureaplasma* (family Mycoplasmataceae, phylum Mycoplasmatota) has been described in the reproductive tract of cattle, through culture [31] and DNA sequencing [32, 33]. It is commonly isolated from cervicovaginal mucosa samples of healthy females; however, it has been associated with reproductive disorders in cows [32, 34, 35]. *Ureaplasma diversum* has been associated with granular vulvitis, endometritis, and reproductive failure, which may be related to its ability to disrupt prostaglandin production by endometrial cells [35–37]. Noteworthy, all cows in the present study were healthy, indicating that those might be opportunistic bacteria that are normally present in the bovine vagina.

The phylum Proteobacteria, which includes *Histophilus* (1.8%) as one of the genera, is related to reproductive disorders and can exist in pathogenic forms, which can result in abortion, mastitis, and granular vulvovaginitis [38, 39]. The results of the present study corroborate the literature, noting the presence of these bacteria in animals that did not present pregnancy after AI. Deng et al. (2019) [40] also aimed to accurately differentiate the vaginal microbiota of pregnant beef heifers and identified the genera *Histophilus*, *Clostridiaceae*, and *Campylobacter* as predictors of pregnancy, with *Histophilus somni* being the most important, which is in agreement with the data from the present study.

The high abundance of *Lactobacillus* spp. in the vaginal microbiota of women during pregnancy may increase reproductive fitness, maintaining a stable microbiota and preventing ascending infections linked to preterm delivery [41, 42]. Although vaginal *Lactobacillus* might reduce the risk of reproductive disorders in humans [1, 19, 43, 44], these bacteria are found in small abundances in the cattle vagina [45, 46]. This was also shown in the present study, in which the genus was not present among the most abundant bacteria. The role of Lactobacillus spp. in the bovine reproductive tract, including methods of microbiota manipulation (i.e. probiotics) deserves further investigation.

### Comparison of NGS methodologies (PacBio *vs*. Illumina)

As expected, an agreement was observed between the two technologies at the phylum level, except for unclassified bacteria, which were identified in greater abundance using the Illumina platform. At the genus level, this agreement was less evident, with Illumina results showing a more similar pattern between samples and PacBio results revealing greater interindividual differences (Fig 3) This information is of importance because although the parameters used to analyze the two technologies were the same, including the reference database used to classify the reads (RDP), the most meaningful results (genus level) might not be as precise and therefore reliable. It was beyond the scope of this study to investigate which of the methods were more accurate in classifying the bovine vaginal bacteria, but it could be assumed that long reads should provide more precise data. Noteworthy, the differences observed between technologies might be due to methodological differences, such as the use of different primers before PCR amplification, as well as DNA degradation caused by storage and shipment for the PacBio sequencing facility.

Long-read sequencing is normally used to investigate the taxonomy of bacteria of interest, but this study supports the fact that it may also better characterize such communities. This is illustrated by the example of the genera *UCG-005* and *UCG-010*, identified as the first most abundant by PacBio, whereas only unclassified Ruminococcaceae, the family to which those genera belong, was present in the Illumina results. These data differ from other studies that compared short reading sequencing (Illumina) with long reads (Nanopore), which stated agreement between the techniques without mentioning the taxonomic level [11].

Only weak and non-significant correlations were observed between alpha diversity indices obtained with the two methods. The significantly higher number of observed ASVs in Illumina results can be explained by the greater number of reads obtained with Illumina sequencing, and consequently a greater number of reads used for the subsampling. In fact, three of the samples had to be excluded from PacBio analysis due to the low yield for reads. The extremely high richness (number of observed species) and estimated number of species (Chao index) is caused by the approach used for data analysis (ASV) and is biologically unrealistic.

The significantly greater number of estimated species obtained by the Chao index for PacBio analysis is indicative of a higher number of unique and rare sequencing reads present in that dataset. This information might be of importance for future studies using a similar approach.

## Conclusions

PacBio sequencing was not able to classify the main bacteria present in the vaginal microbiota of cows at the species level, either because of the poor quality of databanks or because of the presence of unknown organisms. The present study expands on the current knowledge of bovine microbiota, highlighting the need for further efforts to improve the current databanks. The use of more comprehensive techniques, such as shotgun metagenomic sequencing and culturomics, may lead to a better understanding of this environment and the interaction between the bovine vaginal microbiota and its host.

Illumina sequencing yielded a higher number of reads, but PacBio sequencing was able to classify more reads (less unclassified bacteria). The composition of the vaginal microbiota varied according to the sequencing method used, which might complicate the interpretation of results obtained in the majority of the current studies. Larger cohorts are necessary to establish the role of the vaginal microbiota in cow fertility.
